# Nitrogen factor of common carp *Cyprinus carpio* fillets with and without skin

**DOI:** 10.1038/s41598-021-89491-y

**Published:** 2021-05-11

**Authors:** Alena Honzlova, Helena Curdova, Lenka Schebestova, Pavel Bartak, Alzbeta Stara, Josef Priborsky, Anna Koubova, Zdenka Svobodova, Josef Velisek

**Affiliations:** 1 State Veterinary Institute Jihlava, Rantirovska 93/20, 586 01 Jihlava, Czech Republic; 2 Faculty of Fisheries and Protection of Waters, South Bohemian Research Center of Aquaculture and Biodiversity of Hydrocenoses, Research Institute of Fish Culture and Hydrobiology, University of South Bohemia in Ceske Budejovice, Zatisi 728/II, 389 25 Vodnany, Czech Republic

**Keywords:** Chemical biology, Freshwater ecology

## Abstract

Consumer protection against food adulteration and misleading labelling is integrated into EU legislation, but accurate analysis of the meat content of farmed freshwater fish products is not possible because of the lack of established nitrogen factors for farmed common carp. The aim of this study was to determine nitrogen factors for farmed common carp *Cyprinus carpio*. Seven-hundred samples collected in 2018–2019 in three harvest seasons (March/April, Jun/July, and October/November) at seven locations in the Czech Republic were analysed for nitrogen, dry matter, protein, ash, and fat content according to standard ISO methods. The recommended nitrogen factor for fat-free common carp fillet with skin is 3.04 ± 0.13 and, for fillet without skin, 2.95 ± 0.12. Availability of nitrogen factors for common carp can help ensure that consumers are purchasing correctly labelled products.

## Introduction

Instances of food adulteration and penalties for misrepresentation of products are frequently mentioned in historical documents, and meat is a commodity with high potential for adulteration^1,2^. The most common form of adulteration of food is the economically motivated addition or partial substitution of a substance for genuine ingredients to gain a financial advantage^2–4^. Such practices can endanger the health of consumers. Examples include the addition of melamine to milk powder used for infant formula, of Sudan dyes to chili powder and paprika, and of methanol to spirits, as well as the European horsemeat scandal in 2013^1,2,5^. Another example of misleading consumers can be undisclosed addition of water. Some of the farmed species of freshwater fish like common carp are very popular and sought after by consumers and are among the more expensive foodstuffs. This fact can lead the producers to adulteration of food.

Consumer protection against adulteration and misleading food labelling is contained in EU legislation. The Regulation (EU) No. 1169/2011 of the European Parliament and the council on the provision of food information to consumers^6^ includes the requirement for a Quantitative Ingredients Declaration (QUID), which means that processed meat products must be labelled with a statement of the percentage of meat present in the product. Methods of detecting water added to meats have been published for pork^7^, chicken^8^, and seafoods^9^ in European legislation and in standards of Codex Alimentarius. The meat content of fish products is calculated by comparison of the nitrogen content of a particular product with the known fish species nitrogen content. The regulation of meat content in farmed freshwater fish products is not possible because of the absence of established species-specific nitrogen content (nitrogen factor) for farmed fish, with the exception of tilapia^9^.

For this study we selected common carp *Cyprinus carpio,* because Cyprinids represent about 38% of all aquaculture by weight. Common carp is a major farmed species in Asia and European freshwater aquaculture, mainly in central and eastern European countries. Carp contributed ~ 4.67 million metric tons in 2015–2016, accounting for roughly 7.4% of the total global inland fishery production^10–13^. Fisheries of the Czech Republic, Poland, Hungary, and Germany produce 80% of the carp in the European Union, with approximately 10% of the production going into processed carp products^10,13^.

The aim of this study was to determine nitrogen content of common carp *Cyprinus carpio* flesh, the main fish species farmed and processed for foreign and domestic markets in the Czech Republic. Nitrogen factors have the potential for use in establishing meat content of carp products. Determined factors will be used in the control of the fish meat content allows to detect adulteration of fish products by the addition of water.

## Results

The basic nutritional composition of carp fillets with skin and without skin is provided in Table 1.

**Table 1:** Live weight and length of common carp *Cyprinus carpio*, and chemical composition of fillets with and without skin.

Fishery	Season year	Weight (g)	Total length (cm)	Fillet	Dry matter (g/100 g)	Ash (g/100 g)	Fat (g/100 g)	Protein (g/100 g)	N (g/100 g)	Fat–free N (g/100 g)
FFPW USB Vodnany	Autumn 2018	1857.0 ± 195.7 (1365.0–2080.0)	45.6 ± 1.8 (42.0–48.5)	Wisth skin	28.06 ± 2.59^b(B)^ (24.29–33.50)	1.35 ± 0.15^b(A)^ (1.12–1.71)	8.03 ± 2.91^b(A)^ (4.20–14.26)	18.29 ± 0.51^b(B)^ (17.28–19.11)	2.93 ± 0.08^b(B)^ (2.76–3.06)	3.18 ± 0.06^b(A)^ (3.04–3.28)
				Without skin	26.58 ± 1.71^a(A)^* (24.15–30.00)	1.29 ± 0.08^ab(A)^ (1.16–1.46)	6.53 ± 1.87^b(A)^ (4.30–10.20)	17.96 ± 0.40^ab(AB)^ (17.54–18.86)	2.87 ± 0.06^b(B)^ (2.81–3.02)	3.08 ± 0.08^ab(A)^ (2.95–3.19)
	Autumn 2019	2226.5 ± 534.9 (1490.0–3315.0)	51.6 ± 4.24 (45.0–59.0)	With skin	34.09 ± 2.92^b(B)^ (29.17–39.37)	1.26 ± 0.11^ab(A)^ (1.06–1.47)	16.62 ± 4.12^c(B)^ (9.55–23.79)	15.87 ± 0.93^a(A)^ (14.01–17.29)	2.54 ± 0.15^a(A)^ (2.24–2.77)	3.05 ± 0.08^ab(A)^ (2.94–3.21)
				Without skin	30.39 ± 2.62^b(B)^ (26.08–34.60)	1.26 ± 0.18^ab(A)^ (1.06–1.53)	12.52 ± 3.05^c(B)^ (6.69–17.58)	16.03 ± 0.68^a(A)^ (14.74–17.04)	2.56 ± 0.11^a(A)^ (2.36–2.73)	2.93 ± 0.07^ab(A)^ (2.81–3.06)
Blatna	Spring 2018	2297.5 ± 446.5 (1575.0–2930.0)	50.4 ± 2.1 (45.5–53.0)	With skin	27.40 ± 3.87^ab(AB)^ (22.22–36.49)	1.00 ± 0.06^ab(A)^ (0.87–1.10)	9.10 ± 4.68^b(B)^ (3.49–18.57)	17.74 ± 0.52^ab(AB)^ (16.52–18.25)	2.84 ± 0.08^b(B)^ (2.64–2.92)	3.13 ± 0.10^b(B)^ (2.94–3.25)
				Without skin	24.52 ± 2.67^a(A)^ (21.41–30.69)	1.09 ± 0.06^ab(A)^ (0.96–1.16)	4.68 ± 2.55^a(A)^* (2.15–10.69)	17.85 ± 0.53^ab(AB)^ (16.75–18.64)	2.86 ± 0.08^b(B)^ (2.68–2.98)	3.00 ± 0.07^ab(AB)^ (2.91–3.13)
	Summer 2018	1336.5 ± 236.5 (1015.0–1685.0)	40.9 ± 3.0 (36.5–44.5)	With skin	26.50 ± 3.00^a(A)^ (21.33–30.34)	0.97 ± 0.09^a(A)^ (0.87–1.10)	8.02 ± 3.05^b(B)^ (2.08–11.83)	16.69 ± 0.40^ab(AB)^ (16.10–17.49)	2.67 ± 0.06^ab(AB)^ (2.58–2.80)	2.91 ± 0.10^ab(AB)^ (2.71–3.01)
				Without skin	24.16 ± 2.44^a(A)^ (21.00–28.39)	1.03 ± 0.10^ab(A)^ (0.91–1.18)	5.67 ± 2.58^b(B)^ (2.52–10.05)	16.83 ± 0.33^ab(AB)^ (16.24–17.42)	2.69 ± 0.05^ab(AB)^ (2.60–2.79)	2.86 ± 0.06^a(A)^ (2.75–2.95)
	Autumn 2018	1768.0 ± 206.2 (1455.0–2075.0)	46.2 ± 2.0 (44.0–50.0)	With skin	28.37 ± 2.06^b(B)^ (25.12–32.07)	1.21 ± 0.12^ab(A)^ (0.94–1.35)	9.87 ± 2.84^b(B)^ (5.49–14.86)	16.84 ± 0.85^ab(AB)^ (15.43–17.82)	2.69 ± 0.14^ab(AB)^ (2.47–2.85)	2.99 ± 0.08^ab(AB)^ (2.81–3.10)
				Without skin	25.71 ± 1.17^a(A)^* (23.31–27.29)	1.16 ± 0.10^ab(A)^ (1.00–1.35)	7.14 ± 1.84^b(B)^ (3.83–9.86)	16.75 ± 0.67^ab(AB)^ (15.54–17.70)	2.68 ± 0.11^ab(AB)^ (2.49.2.83)	2.89 ± 0.08^a(A)^ (2.76–2.99)
	Spring 2019	1567.5 ± 162.4 (1380.0–1960.0)	43.3 ± 1.4 (42.0–46.5)	With skin	27.61 ± 0.66^b(B)^ (22.80–34.05)	1.12 ± 0.14^ab(A)^ (0.95–1.40)	9.33 ± 3.71^b(B)^ (3.71–17.18)	16.74 ± 0.06^ab(AB)^ (15.78–17.55)	2.68 ± 0.11^ab(AB)^ (2.53–2.81)	2.96 ± 0.10^ab(AB)^ (2.83–3.18)
				Without skin	24.43 ± 2.31^a(A)^* (21.44–28.97)	1.12 ± 0.07^ab(A)^ (1.02–1.27)	5.58 ± 2.29^b(B)^ (2.15–9.37)	16.88 ± 0.58^ab(AB)^ (15.82–17.94)	2.70 ± 0.09^ab(AB)^ (2.53–2.87)	2.86 ± 0.08^a(A)^ (2.76–3.02)
	Summer 2019	1649.0 ± 251.3 (1235.0–2050.0)	44.6 ± 2.1 (42.0–48.0)	With skin	29.55 ± 2.35^b(B)^ (25.70–34.50)	1.02 ± 0.04^ab(A)^ (0.94–1.07)	10.52 ± 2.74^b(B)^ (6.85–16.45)	17.61 ± 0.48^ab(AB)^ (16.45–18.17)	2.82 ± 0.08^b(B)^ (2.63–2.91)	3.15 ± 0.03^b(B)^ (3.09–3.19)
				Without skin	27.06 ± 1.18^ab(AB)^ (24.50–29.11)	1.09 ± 0.05^ab(A)^ (1.01–1.19)	8.12 ± 1.77^b(B)^ (5.11–10.72)	17.60 ± 0.43^ab(AB)^ (16.76–18.27)	2.82 ± 0.07^b(B)^ (2.68–2.92)	3.06 ± 0.05^ab(AB)^ (3.00–3.13)
	Autumn 2019	1987.0 ± 394.5 (1335.0–2450.0)	47.0 ± 3.9 (40.0–51.5)	With skin	31.33 ± 3.10^b(B)^ (25.47–36.86)	0.92 ± 0.06^a(A)^ (0.79–0.98)	13.96 ± 3.73^c(C)^ (7.05–20.47)	15.97 ± 0.80^a(A)^ (14.96–17.80)	2.56 ± 0.13^a(A)^ (2.39–2.85)	2.97 ± 0.09^ab(AB)^ (2.79–3.12)
				Without skin	28.89 ± 3.30^b(B)^ (23.89–34.34)	0.99 ± 0.07^a(A)^ (0.85–1.11)	11.53 ± 3.78^bc(BC)^ (5.52–17.32)	16.05 ± 0.89^a(A)^ (14.39–17.39)	2.57 ± 0.14^a(A)^ (2.30–2.78)	2.90 ± 0.06 ^ab(AB)^ (2.78–2.99)
Chlumec nad Cidlinou	Spring 2018	2108.0 ± 392.4 (1615.0–3010.0)	47.2 ± 3.8 (40.5–53.0)	With skin	35.88 ± 4.38^b(B)^ (28.37–43.72)	0.85 ± 0.24^a(A)^ (0.54–1.36)	18.65 ± 5.40^c(C)^ (11.58–28.14)	15.71 ± 1.00^a(A)^ (13.72–16.89)	2.51 ± 0.16^a(A)^ (2.20–2.70)	3.09 ± 0.12^ab(A)^ (2.91–3.27)
				Without skin	30.83 ± 3.36^b(B)^ (26.24–35.88)	0.97 ± 0.27^a(A)^ (0.46–1.51)	13.80 ± 4.05^c(C)^ (8.53–20.44)	16.01 ± 1.07^a(A)^ (13.99–17.43)	2.56 ± 0.17^a(A)^ (2.24–2.79)	2.97 ± 0.13^ab(A)^ (2.80–3.15)
	Summer 2018	2953.5 ± 658.5 (2045.0–3865.0)	51.2 ± 3.3 (46.0–55.0)	With skin	36.42 ± 2.47^b(B)^ (31.84–40.60)	0.93 ± 0.10^a(A)^ (0.81–1.19)	19.64 ± 2.26^c(C)^ (15.97–24.26)	15.81 ± 0.70^a(A)^ (14.38–16.83)	2.53 ± 0.11^a(A)^ (2.30–2.69)	3.15 ± 0.16^b(A)^ (2.86–3.40)
				Without skin	32.22 ± 1.89^b(B)^ (29.24–35.81)	1.06 ± 0.11^ab(A)^ (0.91–1.34)	14.98 ± 2.61^c(C)^ (10.60–18.88)	15.87 ± 0.68^a(A)^ (14.73–17.18)	2.54 ± 0.11^a(A)^ (2.36–2.75)	2.99 ± 0.12^ab(A)^ (2.73–3.12)
	Autumn 2018	2035.5 ± 216.7 (1485.0–2500.0)	46.7 ± 1.9 (44.0–50.0)	With skin	29.66 ± 3.71^b(B)^ (23.84–35.31)	0.86 ± 0.09^a(A)^ (0.72–1.01)	11.66 ± 4.38^bc(BC)^ (4.01–17.89)	16.56 ± 0.72^ab(AB)^ (15.64–17.67)	2.65 ± 0.11^ab(AB)^ (2.50–2.83)	3.00 ± 0.05^ab(A)^ (2.93–3.12)
				Without skin	27.92 ± 3.51^b(B)^ (21.92–33.39)	0.93 ± 0.11^a(A)^ (0.75–1.08)	9.88 ± 4.20^b(B)^ (2.40–16.08)	16.83 ± 0.82^ab(AB)^ (15.68–17.88)	2.69 ± 0.13^ab(AB)^ (2.51–2.86)	2.99 ± 0.06^ab(A)^ (2.91–3.11)
	Spring 2019	1827.0 ± 204.9 (1475.0–2315.0)	46.5 ± 1.1 (44.5–48.5)	With skin	35.51 ± 3.88^b(B)^ (28.21–39.72)	0.98 ± 0.08^a(A)^ (0.87–1.19)	16.06 ± 4.64^c(C)^ (8.71–23.34)	15.97 ± 0.82^a(A)^ (14.77–17.72)	2.56 ± 0.13^a(A)^ (2.36–2.83)	3.05 ± 0.07^ab(A)^ (2.96–3.20)
				Without skin	28.62 ± 3.59^b(B)^ (22.68–34.74)	1.07 ± 0.06^ab(A)^ (0.98–1.16)	11.00 ± 4.47^bc(BC)^ (4.80–19.13)	16.36 ± 0.77^ab(AB)^ (14.90–17.63)	2.62 ± 0.12^a(A)^ (2.38–2.82)	2.94 ± 0.06^ab(A)^ (2.81–3.03)
	Summer 2019	1128.0 ± 193.0 (755.0–1530.0)	39.7 ± 2.1 (35.0–42.0)	With skin	25.72 ± 2.34^a(A)^ (20.77–28.63)	1.08 ± 0.05^ab(A)^ (1.01–1.16)	6.27 ± 2.56^b(B)^ (0.71–9.42)	18.23 ± 0.59^b(B)^ (17.14–18.87)	2.92 ± 0.09^b(B)^ (2.74–3.02)	3.11 ± 0.12^ab(A)^ (2.86–3.24)
				Without skin	23.54 ± 1.89^a(A)^ (20.21–26.47)	1.16 ± 0.06^ab(A)^ (1.08–1.23)	3.94 ± 1.70^a(A)^* (0.58–6.43)	18.13 ± 0.48^b(A)^ (17.29–18.86)	2.90 ± 0.80^b(B)^ (2.77–3.02)	3.02 ± 0.10^ab(A)^ (2.80–3.17)
	Autumn 2019	2726.0 ± 383.6 (2205.0–3480.0)	49.5 ± 2.0 (44.5–51.5)	With skin	36.16 ± 4.32^b(B)^ (28.86–43.34)	0.85 ± 0.08^a(A)^ (0.70–0.97)	20.06 ± 5.68^d(D)^ (10.86–30.08)	15.34 ± 1.12^a(A)^ (13.54–16.81)	2.45 ± 0.18^a(A)^ (2.17–2.69)	3.07 ± 0.07^ab(A)^ (2.94–3.17)
				Without skin	33.79 ± 4.89^b(B)^ (26.10–44.30)	0.86 ± 0.08^a(A)^ (0.70–0.99)	17.40 ± 5.80^ cd(CD)^ (7.94–29.77)	15.08 ± 1.21^a(A)^ (12.72–16.60)	2.41 ± 0.19^a(A)^ (2.04–2.66)	2.92 ± 0.07^ab(A)^ (2.84–3.06)
Klatovy	Spring 2018	1572.5 ± 133.6 (1335.0–1785.0)	43.1 ± 1.2 (40.5–44.0)	With skin	22.02 ± 1.22^a(A)^ (20.56–24.54)	1.02 ± 0.11^ab(AB)^ (0.72–1.13	2.79 ± 1.62^a(A)^ (0.22–6.57)	17.49 ± 0.36^ab(AB)^ (16.96–18.03)	2.80 ± 0.06^b(B)^ (2.71–2.88)	2.88 ± 0.05^a(A)^ (2.78–2.99)
				Without skin	21.40 ± 0.94^a(A)^ (20.16–23.80)	1.11 ± 0.07^ab(AB)^ (0.97–1.21)	1.68 ± 1.20^a(A)^ (0.31–4.80)	17.84 ± 0.37^ab(AB)^ (17.41–18.50)	2.85 ± 0.06^b(B)^ (2.79–2.96)	2.90 ± 0.05^a(A)^ (2.84–2.98)
	Summer 2018	1751.0 ± 360.4 (1315.0–2455.0)	42.8 ± 2.2 (40.0–46.5)	With skin	28.15 ± 3.32^b(B)^ (22.30–34.49)	0.97 ± 0.05^a(A)^ (0.89–1.04)	9.02 ± 3.49^b(B)^ (2.41–15.92)	17.76 ± 0.78^ab(AB)^ (16.51–18.86)	2.84 ± 0.13^b(B)^ (2.64–3.02)	3.13 ± 0.13^ab(A)^ (2.89–3.30)
				Without skin	24.98 ± 2.73^a(A)^* (21.30–29.59)	1.06 ± 0.08^ab(AB)^ (0.94–1.16)	5.62 ± 2.88^b(B)^ (1.51–10.49)	17.81 ± 0.73^ab(AB)^ (16.51–18.66)	2.85 ± 0.12^b(B)^ (2.64–2.99)	3.02 ± 0.14^ab(A)^ (2.77–3.28)
	Autumn 2018	1250.5 ± 173.6 (1115.0–1705.0)	38.3 ± 1.2 (36.0–40.0)	With skin	34.14 ± 1.83^b(B)^ (30.07–36.76)	1.05 ± 0.11^ab(AB)^ (0.89–1.23)	16.77 ± 1.96^c(C)^ (13.85–21.13)	15.58 ± 0.61^a(A)^ (14.12–16.13)	2.49 ± 0.10^a(A)^ (2.26–2.58)	3.00 ± 0.10^ab(A)^ (2.77–3.11)
				Without skin	30.15 ± 1.88^b(B)^ (26.94–33.11)	1.13 ± 0.10^ab(AB)^ (0.97–1.38)	12.31 ± 2.56^c(C)^ (9.10–17.11)	16.03 ± 0.73^a(A)^ (14.36–17.11)	2.57 ± 0.12^a(A)^ (2.30–2.74)	2.93 ± 0.11^ab(A)^ (2.73–3.08)
	Spring 2019	2880.0 ± 390.3 (2430.0–3515.0)	51.0 ± 2.4 (46.0–54.0)	With skin	27.76 ± 2.63^b(B)^ (22.38–32.34)	1.29 ± 0.31^ab(AB)^ (0.90–1.69)	8.50 ± 2.78^b(B)^ (3.89–14.25)	17.46 ± 0.62^ab(AB)^ (16.62–18.59)	2.79 ± 0.10^b(B)^ (2.66–2.97)	3.05 ± 0.10^ab(A)^ (2.94–3.21)
				Without skin	25.62 ± 2.60^a(A)^* (20.89–31.52)	1.35 ± 0.30^b(B)^ (0.96–1.79)	6.02 ± 2.74^b(B)^ (2.04–13.06)	17.55 ± 0.63^ab(AB)^ (16.72–18.63)	2.81 ± 0.10^b(B)^ (2.68–2.98)	2.99 ± 0.10^ab(A)^ (2.85–3.14)
	Summer 2019	1548.5 ± 321.0 (775.0–2000.0)	45.2 ± 3.8 (35.5–50.0)	With skin	24.25 ± 2.5 ^a(A)^ (21.03–29.62)	1.22 ± 0.11^ab(AB)^ (1.05–1.39)	4.49 ± 2.69^ab(AB)^ (0.87–10.11)	18.19 ± 0.42^b(B)^ (17.42–18.78)	2.91 ± 0.07^b(B)^ (2.79–3.00)	3.05 ± 0.06^ab(A)^ (2.97–3.16)
				Without skin	23.12 ± 1.83^a(A)^ (20.43–26.29)	1.27 ± 0.09^ab(AB)^ (1.11–1.45)	3.73 ± 2.15^a(A)^ (0.69–7.25)	17.77 ± 0.33^ab(AB)^ (17.31–18.31)	2.84 ± 0.05^b(B)^ (2.77–2.93)	2.95 ± 0.06^ab(A)^ (2.82–3.04)
	Autumn 2019	2190.5 ± 285.5 (1565.0–2630.0)	44.4 ± 2.1 (40.0–48.0)	With skin	32.59 ± 3.38^b(B)^ (26.33–37.83)	0.88 ± 0.10^a(A)^ (0.73–1.03)	15.62 ± 4.10^c(C)^ (7.52–21.72)	15.43 ± 0.54^a(A)^ (14.47–16.32)	2.47 ± 0.09^a(A)^ (2.32–2.61)	2.93 ± 0.12^ab(A)^ (2.73–3.20)
				Without skin	30.05 ± 3.67^b(B)^ (23.31–34.54)	0.94 ± 0.10^a(A)^ (0.78–1.08)	13.18 ± 4.18^c(C)^ (5.39–18.16)	15.51 ± 0.72^a(A)^ (14.18–16.62)	2.48 ± 0.12^a(A)^ (2.27–2.66)	2.86 ± 0.14^a(A)^ (2.57–3.04)
Lnare	Spring 2018	1569.5 ± 185.6 (1330.0–1880.0)	41.5 ± 1.8 (38.5–44.5)	With skin	28.70 ± 2.70^b(B)^ (24.68–33.60)	0.98 ± 0.08^a(A)^ (0.86–1.17)	10.38 ± 2.84^b(A)^ (5.40–15.90)	17.16 ± 0.75^ab(A)^ (15.92–17.97)	2.75 ± 0.12^ab(A)^ (2.55–2.88)	3.06 ± 0.10^ab(A)^ (2.91–3.23)
				Without skin	24.91 ± 1.8 ^a(A)^* (21.74–27.58)	1.06 ± 0.07^ab(A)^ (0.97–1.17)	6.09 ± 1.93^b(A)^ (2.98–9.59)	17.41 ± 0.71^ab(A)^ (16.22–18.18)	2.79 ± 0.11^ab(A)^ (2.59–2.91)	2.97 ± 0.09^ab(A)^ (2.76–3.08)
	Summer 2018	1471.5 ± 386.9 (815.0–2230.0)	42.8 ± 2.7 (37.5–46.0)	With skin	29.71 ± 3.58^b(B)^ (22.36–35.67)	1.00 ± 0.09^ab(A)^ (0.89–1.19)	10.90 ± 4.28^b(A)^ (3.05–18.64)	17.14 ± 0.85^ab(A)^ (15.79–18.09)	2.74 ± 0.14^ab(A)^ (2.53–2.89)	3.08 ± 0.06^ab(A)^ (2.99–3.15)
				Without skin	26.31 ± 3.21^a(A)^* (20.69–33.32)	1.10 ± 0.06^ab(A)^ (1.00–1.22)	7.73 ± 3.78^b(A)^ (1.81–16.30)	17.34 ± 0.81^ab(A)^ (15.63–18.33)	2.77 ± 0.13^ab(A)^ (2.50–2.93)	3.01 ± 0.10^ab(A)^ (2.73–3.10)
	Autumn 2018	2981.5 ± 199.5 (2630.0–3380.0)	51.3 ± 2.5 (47.0–55.0)	With skin	30.07 ± 2.44^b(B)^ (25.97–34.29)	1.21 ± 0.13^ab(A)^ (0.96–1.37)	11.18 ± 2.68^bc(B)^ (7.68–16.13)	17.06 ± 0.52^ab(A)^ (16.37–18.45)	2.73 ± 0.08^ab(A)^ (2.62–2.95)	3.08 ± 0.09^ab(A)^ (2.93–3.23)
				Without skin	27.09 ± 2.68^ab(AB)^ (22.71–32.74)	1.22 ± 0.10^ab(A)^ (1.10–1.41)	7.87 ± 3.00^b(A)^ (3.20–14.30)	17.28 ± 0.31^ab(A)^ (16.75–17.81)	2.77 ± 0.05^ab(A)^ (2.68–2.85)	3.00 ± 0.08^ab(A)^ (2.84–3.13)
	Spring 2019	1170.0 ± 84.2 (1075.0–1285.0)	38.9 ± 2.5 (34.0–43.0)	With skin	30.08 ± 2.65^b(B)^ (26.46–34.96)	1.19 ± 0.14^ab(A)^ (0.91–1.41)	12.40 ± 3.25^c(B)^ (6.92–17.55)	16.25 ± 0.72^ab(A)^ (15.36–17.83)	2.60 ± 0.12^a(A)^ (2.46–2.85)	2.97 ± 0.07^ab(A)^ (2.86–3.07)
				Without skin	26.13 ± 2.27^a(A)^* (23.11–30.63)	1.23 ± 0.12^ab(A)^ (1.10–1.45)	8.04 ± 2.88^b(A)^* (3.28–13.56)	16.77 ± 0.71^ab(A)^ (15.66–18.47)	2.68 ± 0.11^ab(A)^ (2.51–2.96)	2.92 ± 0.06^ab(A)^ (2.83–3.06)
	Summer 2019	1800.0 ± 323.3 (1315.0–2305.0)	41.8 ± 3.1 (37.0–47.0)	With skin	30.41 ± 2.16^b(B)^ (27.63–34.25)	0.98 ± 0.13^a(A)^ (0.65–1.13)	13.49 ± 2.24^c(B)^ (10.76–16.81)	15.51 ± 0.49^a(A)^ (14.67–16.18)	2.48 ± 0.08^a(A)^ (2.35–2.59)	2.87 ± 0.09^a(A)^ (2.73–3.05)
				Without skin	26.99 ± 2.13^ab(AB)^ (24.61–31.38)	1.00 ± 0.16^ab(A)^ (0.61–1.27)	9.67 ± 2.49^b(A)^* (6.70–14.81)	15.65 ± 0.50^a(A)^ (14.78–16.26)	2.50 ± 0.08^a(A)^ (2.36–2.60)	2.77 ± 0.10^a(A)^ (2.65–2.94)
	Autumn 2019	1858.5 ± 123.9 (1680.0–2035.0)	44.9 ± 1.5 (43.0–48.0)	With skin	28.96 ± 2.50^b(B)^ (25.61–33.41)	0.91 ± 0.04^a(A)^ (0.85–0.98)	10.83 ± 2.43^b(A)^ (6.80–14.52)	16.82 ± 0.40^ab(A)^ (16.27–17.57)	2.69 ± 0.06^ab(A)^ (2.60–2.81)	3.02 ± 0.08^ab(A)^ (2.90–3.13)
				Without skin	26.97 ± 2.37^ab(AB)^ (23.78–31.76)	0.97 ± 0.06^a(A)^ (0.89–1.09)	8.56 ± 2.36^b(A)^ (4.85–12.12)	16.96 ± 0.45^ab(A)^ (16.13–17.57)	2.71 ± 0.07^ab(A)^ (2.58–2.81)	2.97 ± 0.09^ab(A)^ (2.81–3.07)
Tabor	Spring 2018	2483.5 ± 463.9 (1885.0–3685.0)	50.2 ± 2.6 (47.5–56.5)	With skin	32.63 ± 3.83^b(B)^ (27.24–40.48)	1.02 ± 0.15^ab(AB)^ (0.79–1.28)	15.80 ± 4.16^c(C)^ (9.21–24.14)	16.14 ± 0.71^ab(A)^ (15.16–17.36)	2.58 ± 0.11^a(A)^ (2.43–2.78)	3.07 ± 0.16^ab(AB)^ (2.76–3.28)
				Without skin	30.53 ± 3.85^b(B)^ (25.25–37.79)	1.07 ± 0.21^ab(AB)^ (0.84–1.61)	14.60 ± 4.29^c(C)^ (7.27–22.06)	15.87 ± 0.57^a(A)^ (14.91–16.81)	2.54 ± 0.09^a(A)^ (2.39–2.69)	2.96 ± 0.15^ab(AB)^ (2.70–3.15)
	Summer 2018	1333.8 ± 202.7 (985.0–1650.0)	43.0 ± 2.9 (38.5–47.5)	With skin	28.28 ± 2.39^b(B)^ (24.82–31.89)	1.00 ± 0.11^ab(AB)^ (0.84–1.13)	9.37 ± 3.15^b(B)^ (4.83–14.19)	17.58 ± 0.85^ab(A)^ (16.27–18.22)	2.81 ± 0.14^b(B)^ (2.60–3.01)	3.10 ± 0.11^ab(AB)^ (2.82–3.22)
				Without skin	25.29 ± 2.01^a(A)^* (22.49–28.16)	1.11 ± 0.13^ab(AB)^ (0.92–1.32)	6.04 ± 2.72^b(B)^ (1.77–10.64)	17.63 ± 0.74^ab(A)^ (16.40–18.68)	2.82 ± 0.12^b(B)^ (2.62–2.99)	3.00 ± 0.09^ab(AB)^ (2.84–3.10)
	Autumn 2018	2245.0 ± 375.2 (1545.0–2800.0)	50.0 ± 3.1 (45.5–54.5)	With skin	32.74 ± 2.86^b(B)^ (26.29–35.59)	1.30 ± 0.21^b(B)^ (0.98–1.65)	13.96 ± 3.25^c(C)^ (6.60–17.61)	17.12 ± 0.57^ab(A)^ (15.97–17.89)	2.74 ± 0.09^ab(AB)^ (2.56–2.86)	3.19 ± 0.10^b(B)^ (3.02–3.32)
				Without skin	31.84 ± 2.91 ^b(B^ (25.11–34.67)	1.26 ± 0.20^ab(AB)^ (0.94–1.55)	12.98 ± 3.38^c(C)^ (5.67–17.20)	16.87 ± 0.65^ab(A)^ (15.49–17.61)	2.70 ± 0.10^ab(AB)^ (2.48–2.82)	3.10 ± 0.09^ab(AB)^ (2.99–3.26)
	Spring 2019	2637.0 ± 615.0 (1960.0–2085.0)	52.2 ± 2.9 (47.5–58.5)	With skin	23.84 ± 2.46^a(A)^ (16.34–18.36)	1.08 ± 0.07^ab(AB)^ (1.01–1.26)	4.73 ± 2.77^a(A)^ (0.56–9.90)	17.56 ± 0.63^ab(A)^ (16.34–18.36)	2.81 ± 0.10^b(B)^ (2.61–2.94)	2.95 ± 0.08^ab(AB)^ (2.84–3.06)
				Without skin	22.64 ± 1.89^a(A)^ (20.27–26.86)	1.09 ± 0.06^ab(AB)^ (1.03–1.22)	3.52 ± 2.17^a(A)^ (0.51–8.64)	17.30 ± 0.60^ab(A)^ (16.46–18.35)	2.77 ± 0.10^ab(AB)^ (2.63–2.94)	2.87 ± 0.09^a(A)^ (2.71–3.03)
	Summer 2019	1755.5 ± 206.1 (1445.0–2085.0)	47.8 ± 2.2 (44.0–51.5)	With skin	25.96 ± 4.41^a(A)^ (20.14–35.80)	1.02 ± 0.13^ab(AB)^ (0.77–1.21)	7.91 ± 5.87^b(B)^ (1.03–20.61)	16.88 ± 1.59^ab(A)^ (13.81–18.64)	2.70 ± 0.26^ab(AB)^ (2.21–2.98)	2.93 ± 0.13^ab(AB)^ (2.70–3.08)
				Without skin	24.61 ± 3.88^a(A)^ (20.15–33.67)	1.09 ± 0.11^ab(AB)^ (0.89–1.27)	6.89 ± 5.20^b(B)^ (1.37–18.85)	16.42 ± 1.66^ab(A)^ (13.46–18.21)	2.63 ± 0.27^ab(AB)^ (2.15–2.91)	2.82 ± 0.17^a(A)^ (2.51–3.00)
	Autumn 2019	1613.5 ± 368.1 (1260.0–2315.0)	43.4 ± 3.0 (40.0–50.0)	With skin	32.78 ± 1.88^b(B)^ (29.36–35.44)	0.84 ± 0.11^a(A)^ (0.73–1.01)	15.39 ± 2.33^c(C)^ (11.57–19.34)	16.07 ± 0.41^ab(A)^ (13.37–16.64)	2.57 ± 0.07^a(A)^ (2.46–2.66)	3.04 ± 0.05^ab(AB)^ (2.94–3.11)
				Without skin	30.12 ± 2.01^b(B)^ (26.67–33.24)	0.92 ± 0.10^a(A)^ (0.80–1.11)	12.35 ± 2.49^c(C)^ (8.39–16.48)	16.35 ± 0.38^ab(A)^ (15.59–16.96)	2.62 ± 0.06^a(A)^ (2.49–2.71)	2.98 ± 0.05^ab(AB)^ (2.92–3.08)
Hodonin	Spring 2019	2084.0 ± 235.3 (1770.0–2645.0)	49.3 ± 1.8 (45.5–51.5)	With skin	31.47 ± 5.54^b(A)^ (25.94–43.91)	0.97 ± 0.10^a(A)^ (0.81–1.21)	13.86 ± 7.00^c(B)^ (13.86–28.58)	16.30 ± 1.59^ab(A)^ (13.48–18.21)	2.61 ± 0.25^a(A)^ (2.16–2.91)	3.03 ± 0.13^ab(A)^ (2.66–3.16)
				Without skin	28.81 ± 5.68^b(A)^ (23.04–42.91)	1.01 ± 0.10^ab(A)^ (0.83–1.21)	11.61 ± 7.41^bc(AB)^ (4.28–28.72)	16.10 ± 1.95^ab(A)^ (12.65–18.37)	2.56 ± 0.31^a(A)^ (2.02–2.94)	2.89 ± 0.15^ab(A)^ (2.52–3.07)
	Summer 2019	2004.5 ± 334.7 (1585.0–2765.0)	46.1 ± 2.0 (42.0–50.0)	With skin	29.26 ± 2.59^b(A)^ (25.73–34.02)	0.75 ± 0.09^a(A)^ (0.55–0.88)	10.57 ± 3.63^b(A)^ (6.04–17.06)	17.31 ± 0.78^ab(A)^ (16.13–18.60)	2.77 ± 0.12^ab(AB)^ (2.58–2.98)	3.10 ± 0.07^ab(A)^ (2.98–3.23)
				Without skin	27.26 ± 1.70^b(A)^ (24.57–30.51)	0.81 ± 0.07^a(A)^ (0.66–0.92)	9.10 ± 1.89^b(A)^ (5.82–12.16)	17.47 ± 0.29^ab(A)^ (17.12–17.92)	2.80 ± 0.05^b(B)^ (2.74–2.87)	3.08 ± 0.05^ab(A)^ (2.96–3.13)
	Autumn 2019	2465.0 ± 343.0 (1765.0–3120.0)	49.3 ± 3.8 (43.0–56.0)	With skin	31.53 ± 7.43^b(A)^ (21.77–47.61)	0.94 ± 0.19^a(A)^ (0.55–0.19)	14.92 ± 8.56^c(B)^ (5.05–34.09)	15.80 ± 1.68^a(A)^ (11.42–17.22)	2.53 ± 0.27^a(A)^ (1.83–2.76)	2.97 ± 0.12^ab(A)^ (2.77–3.14)
				Without skin	29.85 ± 7.54^b(A)^ (20.30–46.07)	1.02 ± 0.15^ab(A)^ (0.68–1.21)	16.05 ± 9.01^c(B)^ (3.41–33.24)	15.67 ± 1.74^a(A)^ (11.53–17.84)	2.51 ± 0.28^(A)^ (1.84–2.85)	2.88 ± 0.08^a(A)^ (2.76–2.99)

Data are mean ± standard deviation (range), n = 10. Different lower-case superscripts indicate significant (p < 0.01) differences among the locations. Different upper-case superscripts indicate significant (p < 0.01) differences among seasons in a localation.

*Denotes significant differences between fillets with and fillets without skin at a single sampling (p < 0.01).

In this study, the nitrogen content was significantly lower (*p* ˂ 0.01) in carp fillets with and without skin from FFPW USB Vodnany (autumn 2019), Blatna (autumn 2019), Chlumec nad Cidlinou (spring 2018, summer 2018, spring 2019, autumn 2019), Klatovy (autumn 2018, 2019), Lnare (summer 2019), Tabor (spring 2018, autumn 2019), Hodonin (spring 2019, autumn 2019) and fillets with skin from Lnare (spring 2019) compared to fillets with skin from Tabor in spring 2019, without skin from Hodonin (summer 2019), and fillets with and without skin from FFPW USB Vodnany (autumn 2018), Tabor (summer 2018), Blatna (spring 2018, summer 2019), Chlumec nad Cidlinou (summer 2019), and Klatovy (spring 2018, 2019, and summer 2018, 2019). We did not find significant differences (*p* ˃ 0.05) in nitrogen content between carp fillets with skin and fillets without skin from a single fish.

In this study, the fat-free nitrogen content was significantly lower (*p* ˂ 0.01) in fillets with and without skin from Klatovy (spring 2018) and Lnare (summer 2019) and in fillets without skin from Blatna (summer 2018, autumn 2018, spring 2019), Klatovy (autumn 2019), Tabor (spring and summer 2019), Hodonin (autumn 2019) compared to fillets with skin from FFPW USB Vodnany in autumn 2018, Blatna in spring 2018 and summer 2019, Chlumec nad Cidlinou in summer 2018, and Tabor in autumn 2018. We did not find significant differences (*p* ˃ 0.05) between carp fillets with and without skin in fat-free nitrogen content from an individual fish.

**Figure 1:**
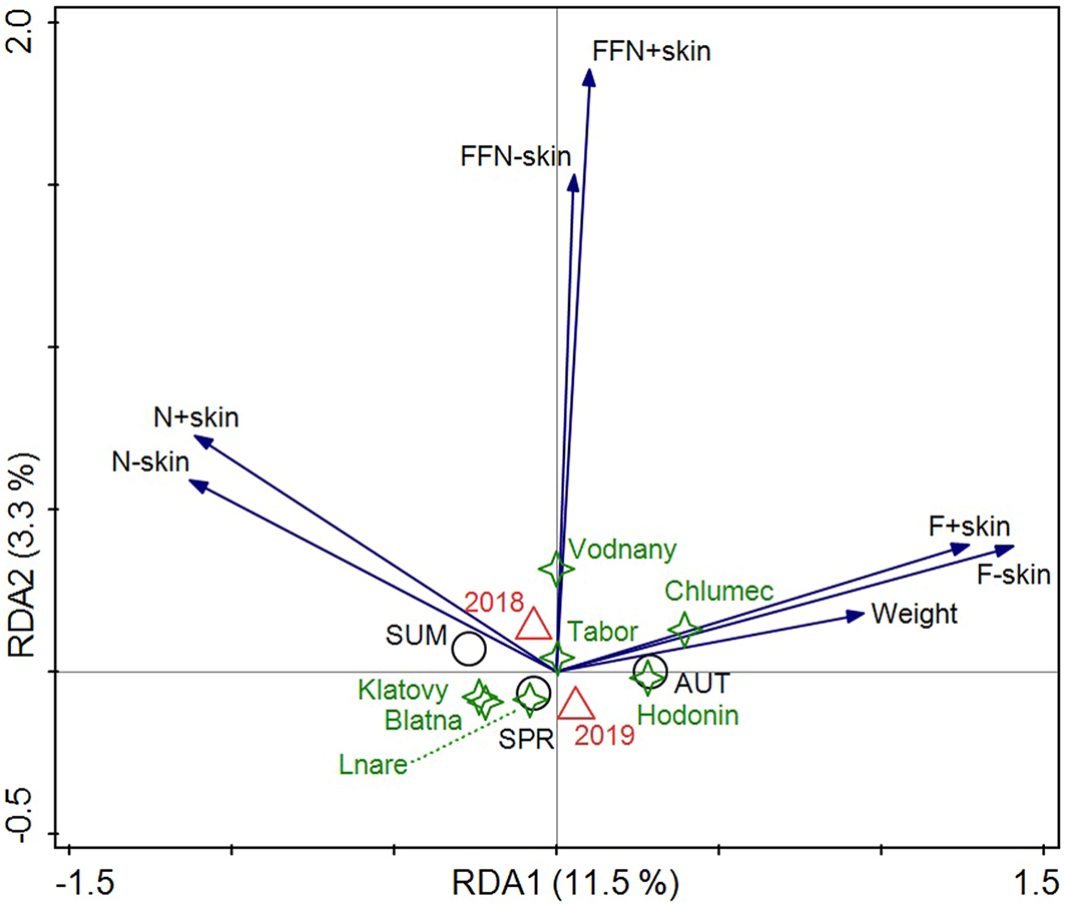
Ordination plot generated by Redundancy analysis (RDA) of functional traits (explained data) and company, sampling year and season as categorial data. Functional traits are displayed by arrows and categorial data by centroids. N + skin—nitrogen in filet with skin, N-skin—nitrogen in filet without skin, FFN + skin—fat-free N in filet with skin, FFN-skin—fat-free N in filet without skin, SPR—spring, SUM—summer, AUT—autumn.

Redundancy analysis using tree categorial variables produced canonical scores corresponding to axes constrained by explanatory variables (Fig. 1). Four canonical axes explaining the variance of response data constrained by categorial data accounted together only 15.7% of the total variance. The first canonical axis explained 11.5% of the total variance. The type of company (fishery), year and season of sampling were not found as strong explanatory factors explaining the variability of samples (permutation test, pseudo-F = 7.01, p = 0.002). This analysis also showed that functional traits of fish filets with and without skin closely correlated together regardless of the type of parameter. There were found the negative correlations between fat and nitrogen content parameters. Fat-free N functional trait were not negatively correlated with nitrogen nor fat contents parameter. The fat content positively correlated with weight of fish.

The established nitrogen factors for common carp fillets with and without skin determined by the Kjeldahl method are 2.68 ± 0.19, 2.69 ± 0.18, respectively. The established fat-free nitrogen factors for common carp fillets with and without skin determined by Kjeldahl method are 3.04 ± 0.13, 2.95 ± 0.12, respectively.

## Discussion

The control of products from common carp for the content of meat is not possible due to absence of nitrogen factors. As far as what is known, there is no information in the literature on the nitrogen factors determined by Kjeldahl method for common carp in fillets with skin and without skin. Sufficient number of samples, 350 of fillets with skin and the same number of fillets without skin of carp common, were analysed for basic chemical composition, dry matter, protein, fat and ash to determine variations between fishery farm (locality), harvesting season, years and fillets with skin and without skin.

The basic nutrient values obtained in this study are similar to those reported in 2003 for fillets with skin of four strains of carp^14^ and with data from 2005 for muscle of 3-old-year carp of five strains^15^. The water percentage decreases, and the fat and protein percentages increase, with increasing body weight and length, whereas the ash percentage remains fairly constant. Our findings are in general agreement with those reported for other type of carp (Asian carp including bighead carp *Hypophthalmichthys nobilis*, silver carp *Hypophthalmichthys molitrix* and grass carp *Ctenopharyngodon idella*)^16–19^ and other fish species^20–23^.

The fat content of fillets with skin varied from 2.79 to 20.06 g/100 g and, for fillets without skin, from 1.68 to 17.40 g/100 g. In this study found the negative correlations between fat and nitrogen content parameters. Fat-free N functional trait were not negatively correlated with nitrogen nor fat contents parameter. The fat content positively correlated with weight of fish. Fat content influences the relative content of other analysed components. Calculation of the fat-free nitrogen factor can reduce the greatest impact of fat variation^24^. The standard deviations of nitrogen factors in fat-free fillets with and without skin are lower than those in fillets with fat.

We suggest that the QUID in farmed carp products should be based on the nitrogen calculation of defatted carp content using a fat-free nitrogen factor. The overall meat content is obtained by adding the determined fat content to the defatted carp content (Table 2). The meat content was calculated for five carp fillets with skin and five without skin with the smallest content of fat and for five carp fillets with skin and five without skin showing with the highest fat content of tested samples. The meat content was calculated based on nitrogen factor N_f_ (total fillet) and the fat-free nitrogen factor N_ff_. The results demonstrated a lesser effect on the calculated meat content when using the fat free nitrogen factor.

**Table 2:** Meat content calculated based on recommended nitrogen factors for common carp *Cyprinus carpio*.

Fat content (g/100 g)	N_f_	Meat content_Nf_ (g/100 g)	N_ff_	Meat content_Nff_ (g/100 g)
**Fillet with skin**				
2.79	2.68	103.9	3.04	94.4
4.49		108.3		100.0
4.73		104.5		96.8
6.27		108.7		102.2
7.91		100.7		96.6
16.62	2.68	94.5	3.04	99.9
16.77		92.5		98.3
18.65		93.3		100.9
19.64		94.4		102.8
20.06		91.6		100.9
**Fillet without skin**				
1.68	2.69	105.5	2.95	97.9
3.52		102.4		96.8
3.73		105.4		99.8
3.94		107.6		101.1
4.68		105.5		100.9
13.80	2.69	95.2	2.95	100.6
14.60		94.4		100.7
14.98		94.2		100.9
16.05		93.2		101.1
17.40		89.4		98.9

Meat content_Nf_ = N × 100/N_f_ (calculated based on nitrogen factor N_f_ (total fillet).

Meat content_Nff_ = DCC (defatted meat content) + fat [N × 100/N_ff_ + F] (calculated based on the nitrogen factor N_ff_ (fat-free fillet).

The established nitrogen factor allows a QUID label stating the fish content of the product, in compliance with EU legislation Regulation No. 1169/2011 of the European Parliament and of the Council on the provision of food information to consumers^6^. Using the recommended nitrogen factors, it is possible to calculate the carp content^9^ for labelling carp products and to detect undisclosed addition of water to the products. The established fat-free nitrogen factors for common carp fillets with and without skin determined by Kjeldahl method are 3.04 ± 0.13, 2.95 ± 0.12, respectively. The recommended nitrogen factors for calculation of the meat content in farmed carp products are the fat-free nitrogen factors because of variation of fat content in fish products. There are limitations to the use of nitrogen factors. They are expressed as mean values with standard deviations, and, when deciding whether declarations of meat or fish content are fulfilled, it is important to bear in mind potential variation (effects of season, weight, fishery location, nutritional status) and to apply the recommended^9^ variation value of ± 10%. The applying of these factors in the control of the fish meat content allows to detect adulteration of common carp products by the addition of water.

Given the fact of small differences in the nutrient values for common carp and other species of carp, it is appropriate to supplement the analysis of the fish meat content with PCR analysis of fish species^25^.

## Materials and methods

### Fish and experimental protocol

Three-hundred-fifty market-size (755–3865 g) common carp *Cyprinus carpio* were obtained from six sources at various times of year to for effects of variation in rearing conditions. The weight of collected carp corresponded to the weight of carp normally delivered to the market. Fish were obtained from the Faculty of Fisheries and Protection of Waters of the University of South Bohemia in Ceske Budejovice (FFPW USB), Vodnany and the fisheries Chlumec nad Cidlinou, Blatna, Hodonin, Klatovy, Lnare, and Tabor. Ten fish were collected from each fishery at the spring (March/April), summer (June/July), and autumn harvests (October/November) in 2018 and 2019. Carp were transported live to the laboratory of the FFPW, killed by a blow to the head, weighed, measured, and filleted. Two fillets, one with skin removed, from each fish were individually vacuum packed, immediately frozen, and stored at − 32 °C until chemical analysis.

### Ethics approval

All the methods used in the present study followed relevant guidelines and regulations. Also, the competent authority (Ethical Committee for the Protection of Animals in Research of the University of South Bohemia, FFPW Vodnany) approved the fish sampling and protocols of the present study and reporting herein follows the recommendations in the ARRIVE guidelines.

### Chemical analysis

Seven-hundred carp fillets were analysed for basic nutritional composition, dry matter, protein, fat, and ash. All samples were homogenized by grinding before analysis.

The determination of dry matter followed ISO 1442:1997 Meat and meat products—Determination of moisture content (Reference method)^26^. The homogenized samples were dried with sand to constant weight at 103 ± 2 °C in a laboratory oven (Memmert UE 500, Memmert GmbH + Co. KG, Germany).

The determination of ash was based on the standard ISO 936:1998 Meat and meat products—Determination of total ash^27^. The homogenized samples were burned in a muffle furnace (Nabertherm A11/HR, Nabertherm GmbH, Germany) at 550 ± 25 °C to a grey-white colour.

The determination of total fat was based on the standard ISO 1443:1973 Meat and meat products—Determination of total fat content^28^. The homogenized samples were hydrolysed by hydrochloric acid, and fat was extracted by light petroleum in SOXTEC 2050 (FOSS Headquarters, Denmark).

The determination of nitrogen used the Kjeldahl method based on the standard method ISO 937:1978 Meat and meat products—Determination of nitrogen content (Reference method)^29^. The homogenized samples were digested by sulphuric acid and a catalyser in a KjelROC Digestor 20 (OPSIS AB, Sweden) digestion unit at 420 ± 10 °C. Organically bound nitrogen was measured on the KJELTEC 8400 with KJELTEC sampler 8420 (FOSS Headquarters, Denmark). Calculation of protein content from nitrogen used the conversion factor for meat of 6.25.

All analysis of dry matter, ash, and total fat were performed in duplicate and analysis of nitrogen (protein) was performed in triplicate for each sample.

Calculation of fat-free nitrogen (N_ff_) in g/100 g used the formula^24^:

$$ N_{ff} = \frac{{100 \times N { }}}{{100 - F { }}}. $$

This formula was applied to nitrogen (N) and fat (F) content for all samples, providing a fat-free nitrogen value for each sample.

Fish meat content calculated based on nitrogen factor N_f_ (total fillet) in g/100 g used the formula^9^:

$$ Fish \;content_{Nf} = \frac{N \times 100}{{N_{f} }}. $$

Fish meat content calculated based on fat-free nitrogen factor (N_ff_) and DCC (defatted carp content) in g/100 g used formulas^11^:

$$ Fishc\; content_{Nff} = DCC + F, $$

$$ DCC = \frac{N \times 100}{{N_{ff} }}. $$

### Statistical analysis

Kolmogorov–Smirnov and Bartlett’s tests were applied to assess normal distribution data and the homoscedasticity of variance, respectively. A two-way ANOVA and Tukey’s test was conducted to analyse effects of season, weight, fishery, and difference between fillets with and without skin. The significance level was set at *p* < 0.05. Data were expressed as the mean ± SD values and range. Analysis was performed using STATISTICA v. 12.0 for Windows (STATSOFT, Inc.).

Redundancy analysis (RDA) with functional traits as response variables and company, sampling year and season as categorial (explanatory) data was applied to describe the differences among sample distribution. The ordination plots were produced using Canoco, Windows release, 5.10 version (Biometris, the Netherlands, and Petr Šmilauer, Czech Republic).

## Data Availability

The data that support the findings of this study are available from the corresponding author upon reasonable request.
